# Who are the vulnerable, and how do we reach them? Perspectives of health system actors and community leaders in Kerala, India

**DOI:** 10.1186/s12889-023-15632-9

**Published:** 2023-04-24

**Authors:** Jaison Joseph, Hari Sankar, Gloria Benny, Devaki Nambiar

**Affiliations:** 1 The George Institute for Global Health, New Delhi, India; 2 Faculty of Medicine, University of New South Wales, Sydney, Australia; 3 Prasanna School of Public Health, Manipal Academy of Higher Education, Manipal, India

**Keywords:** Vulnerable Population, Health Equity, Sex Differences, Universal Health Coverage, Primary Health Care, Health Systems, Primary Care Cost, Primary Care Utilization

## Abstract

**Background:**

Among the core principles of the 2030 agenda of Sustainable Development Goals (SDGs) is the call to Leave no One behind (LNOB), a principle that gained resonance as the world contended with the COVID-19 pandemic. The south Indian state of Kerala received acclaim globally for its efforts in managing COVID-19 pandemic. Less attention has been paid, however, to how inclusive this management was, as well as if and how those “left behind” in testing, care, treatment, and vaccination efforts were identified and catered to. Filling this gap was the aim of our study.

**Methods:**

We conducted In-depth interviews with 80 participants from four districts of Kerala from July to October 2021. Participants included elected local self-government members, medical and public health staff, as well as community leaders. Following written informed consent procedures, each interviewee was asked questions about whom they considered the most “vulnerable” in their areas. They were also asked if there were any special programmes/schemes to support the access of “vulnerable” groups to general and COVID related health services, as well as other needs. Recordings were transliterated into English and analysed thematically by a team of researchers using ATLAS.ti 9.1 software.

**Results:**

The age range of participants was between 35 and 60 years. Vulnerability was described differentially by geography and economic context; for e.g., fisherfolk were identified in coastal areas while migrant labourers were considered as vulnerable in semi-urban areas. In the context of COVID-19, some participants reflected that everyone was vulnerable. In most cases, vulnerable groups were already beneficiaries of various government schemes within and beyond the health sector. During COVID, the government prioritized access to COVID-19 testing and vaccination among marginalized population groups like palliative care patients, the elderly, migrant labourers, as well as Scheduled Caste and Scheduled Tribes communities. Livelihood support like food kits, community kitchen, and patient transportation were provided by the LSGs to support these groups. This involved coordination between health and other departments, which may be formalised, streamlined and optimised in the future.

**Conclusion:**

Health system actors and local self-government members were aware of vulnerable populations prioritized under various schemes but did not describe vulnerable groups beyond this. Emphasis was placed on the broad range of services made available to these “left behind” groups through interdepartmental and multi-stakeholder collaboration. Further study (currently underway) may offer insights into how these communities – identified as vulnerable – perceive themselves, and whether/how they receive, and experience schemes designed for them. At the program level, inclusive and innovative identification and recruitment mechanisms need to be devised to identify populations who are currently left behind but may still be invisible to system actors and leaders.

## Introduction

The core aim of the 2030 agenda of Sustainable Development Goals (SDGs) is to bring in transformation through Sustainable Development which requires nations to Leave no One behind (LNOB) [1]. Populations left behind are defined as being “at greater risk of poor health status and healthcare access, who experience significant disparities in life expectancy, access to and use of healthcare services, morbidity and mortality” [2]. These populations sometimes experience multiple morbidities which results in complex health care needs which are further exacerbated by intersecting deleterious social and economic conditions [2]

Globally, each nation has the prerogative to define “left behind” groups or communities based on the social, economic, cultural and political factors, which in turn may vary across geographies subnationally [3]. In India, groups face vulnerability or marginalization on the basis of age, disability, socio-economic status, which in turn restricts the access of these communities to health and healthcare [4]. Groups that are officially considered vulnerable in India according to the country’s main think tank, the NITI Aayog, include persons who are classified as those in Scheduled Castes (SCs), Scheduled Tribes (STs), Other Backward Classes (OBCs), Economically Backward Classes (EBCs), Religious Minorities, Nomadic, Semi-Nomadic and De-Notified Tribes (NT, SNT & DNTs), people who work in sanitation, known in Hindi as *Safai karmacharis* (SKs), Senior Citizens/ the elderly, Transgendered persons, Persons engaging in Substance Abuse, as well as those who are destitute and involved with begging[4–6]These population subgroups are prioritised for various government welfare schemes. Across the country, participation of under-represented groups in planning an decision-making is instituted through affirmative action: SC, ST and Other Backward Classes (OBCs) are provided reservations in public service.

In the health domain, Below Poverty Line (BPL) households are covered under Ayushman Bharat Pradhan Mantri Jan Arogya Yojana (AB-PMJAY) providing insurance coverage in the amount of 500,000 INR (~ 6,050 USD) per family for secondary and tertiary care hospitalization expenditure through empanelled health care providers [7, 8]. In the Southern Indian state of Kerala, Ayushman Bharat benefits are extended to a broader beneficiary group, comprising Mahatma Gandhi National Rural Employment Guarantee Act (MGNREGA) households, households of unorganized workers and additional population subgroups recognised as facing disadvantage by the state.

Kerala has the lowest level of multidimensional poverty according to the NITI Aayog, which suggests that the population of “vulnerable” may be relatively lower in this setting [9]. Overall, this bears out: the state’s development pattern also indicates relatively low inequalities in health and education outcomes [10]. The state nonetheless takes seriously the process of identifying and catering to “vulnerable” population groups. It has a range of programmes for people recognised as having Scheduled Caste (SC) and Scheduled Tribe (ST) status, women, children, elderly and persons living with disabilities [11]. We identified no less than around 35 schemes and population-specific programs introduced by the state in the past half decade to support groups facing disadvantage: these include earmarked funds, subsidy schemes, as well as reservations in education and employment [3, 12]. Health programs have also been put in place by non-health departments and agencies. For example, the Scheduled Tribes Development Department implements many programs to address the general healthcare needs of tribal populations, which include allopathic health care institutions, medical reimbursement through hospitals, a tribal relief fund for emergency expenditure, assistance for sickle-cell anaemia patients, assistance to traditional tribal healers and mobile medical units [13]. One of the objectives of the Health and Family Welfare Department’s recently launched Aardram mission was to improve access of marginalized/vulnerable populations to comprehensive health services [14]. The state is also implementing free health insurance scheme called “Awaz” for interstate migrant workers, covering Rs.15,000/- (~ 181.82 USD) for medical treatment per year and an amount Rs.200,000/- Lakhs (﻿~ 2424 USD) for accident deaths [15]

Although the state has several welfare measures and schemes to improve healthcare access for vulnerable groups, challenges remain. For one, impoverishment due to health is a major barrier that disproportionately affects those already facing marginalisation: such groups cannot rely on the public sector for services and end up impoverished due to health expenditures in the private sector [16]. In fact, high Out-of-Pocket-Expenditure (OOPE) and rising health care cost for hospitalization have resulted in reducing health seeking [17]. Vulnerabilities therefore, are changing almost continuously. This makes the task of identifying vulnerable groups difficult – given the dynamic, complex, historically, and contextually contingent nature of vulnerability [18]. And yet, both global and national goals call for identification, responses and monitoring of outcomes in these population groups [1, 19].

As part of a larger health systems study, we placed emphasis on how vulnerability is defined in the state, and how vulnerabilities are addressed through schemes and equity-oriented reforms introduced in the state. It is important to understand the perspective of primary care health system actors on vulnerability and who are vulnerable, as they are at the forefront of delivering essential health care services and identification and catering to the needs of vulnerable population. Such an exercise has been carried out, for example in other regions with the support of the World Health Organization, [20]. as well as in other projects focused on equity integration in health programming and planning [21–23]. Barring a rare example published in 2015 [24], we were not able to identify such initiatives or studies in the Indian context, particularly ones that viewed “vulnerability” and efforts at inclusion from an implementer’s perspective. Seeking to fill this gap, we undertook a qualitative analysis of perspectives from Kerala’s health system actors, local self-government representatives and community leaders involved with Primary Healthcare Reforms (PHCR) in Kerala about their definitions and understandings of who is vulnerable in the state, what is being done to address their vulnerabilities, both within and outside of the context of COVID-19.

## Methods

This study is the qualitative component of a larger health system research study in Kerala; our detailed methodology is reported elsewhere[25]. In summary, Kerala’s 14 districts were grouped into four categories using principal components analysis, using indicators from the fourth round of the National Family Health Survey (NFHS) (2015–16) [26]. One district was randomly selected from each group, within which catchment areas served by two randomly selected primary health facilities (one recently upgraded by Aardram and one slated for later upgradation) were also randomly selected.

In-depth interviews (IDIs) were carried out in the four selected districts between July and October 2021. Participants for this study were staff from two primary healthcare facilities per district and elected representatives from their corresponding Local Self Governments (LSGs). We adopted purposive criterion sampling technique for the selection and recruitment of study participants. For the identification and selection of participants we employed a two-pronged strategy. As an initial step we line-listed the potential health system actors (HSAs) and community leaders who could be part of this study. From each facility we enrolled HSAs including medical and public health staff, community leaders and Local Self Government representatives to obtain a comprehensive HSAs perception of vulnerable population their area. Medical and public health staff included, Medical Officer (MO), Staff Nurse/Nursing Officer, Health Inspector (HI), Junior Health Inspector (JHI), Public Health Nurse (PHNs), Junior Public Health Nurse (JPHNs), Palliative Care Nurse and Accredited Social Health Activists (ASHAs). Community members eligible for recruitment included Panchayat Presidents and Vice Presidents, Health Standing Committee member and Ward Members. We identified additional community leaders from these areas through the HSAs, LSG members and non-governmental organizations to capture the perspective of the community. On an average we enrolled 10 HSA per facility, a total of 83 HSAs were contacted for this study and three of them could not participate due to their busy schedule.

The Institutional Ethics Committee of the George Institute for Global Health (Project Number 05/2019) issued ethical approval for this study. In each facility area, in-depth interviews for this study were carried out by three researchers trained in qualitative research methods (HS, JJ & GB). The research team comprised of two male research fellows and a female research assistant and was supervised by a senior health systems researcher (DN). Administrative approval was taken from the Department of Health and Family Welfare, Government of Kerala. The team met the District Medical Officers (DMO) of four districts, shared the departmental permissions, outlined the study objectives, and shared findings of an earlier primary survey carried out in the same catchment areas. After the permissions were issued from the DMOs, the team of three researchers (HS, GB, JJ) took appointments with Medical Officers and briefed them about the study and sought their permission for conducting IDIs with the staff under their institutions. Further, each of the HSAs were met in person and appointments for interviews were sought based on their convenience. As per their convenience IDIs were carried out in-person or through online platforms (i.e. Zoom). For carrying out the IDIs with LSG representatives, the team met with the panchayat presidents of the respective LSGs and briefed on the purpose of study and sought their permission to carry out the IDIs with other identified LSG members. Community leaders were contacted over phone, to brief them on the purpose of the study and as per their convenience the researcher met them in person to carry out the interviews.

All the participants were handed over with a hard copy of the topic guides and Participant Information Sheet (PIS) in English and Malayalam before the in-person interviews. Each participant’s signed informed consent was taken for participating in the study and for recording interviews. For those interviews conducted over online platforms, a soft copy of the topic guide, PIS and consent form were shared in advance with the participants. Before commencing the interview, the participants shared the dully signed consent form with the researchers. Malayalam was the medium of conversation and each of the IDIs lasted between 20 and 60 min. To obtain context and perspectives of HSAs in various capacities and geographies pertaining to each of the study sites across four districts the interviews with all the pre-set list of participants were completed even though achieving early data saturation was reached with some of the study topics.

Three participants could not participate in the interview due to their busy schedules and after multiple failed attempts to schedule, we decided to remove them from the study. All IDIs were recorded; interview recordings and field notes were stored and secured in a password protected database after the completion of each interview and were accessible only to the research team members. Recordings were transliterated into English by a third-party agency empanelled by The George Institute for Global Health, India, which signed confidentiality agreements prior to accessing data. All the transliterated transcripts were reviewed by a three-member research team to ensure quality.

Transliterated transcripts were thematically analysed using ATLAS.ti 9 software by a four-member research team (DN, HS, JJ, GB). An inductive approach was used: the thematic structure and code book were finalized after multiple discussions among the four-member team. Finally, the coded manuscripts from the team members were merged using ATLAS.ti 9 software. Codes of interest for this analysis were indexed and themes consolidated based on further discussions and core questions of interest (i.e., who is left behind? How are they reached? and impact of COVID-19 among those left behind). A narrative was then constructed around these questions and compiled by the lead author with inputs, edits, and review by other authors.

## Results

### Participant characteristics

Data for a total of 80 participants was included in the study, of which more than half (60%) were women (see Table 1). From this group of participants, we received information on who they considered was being left behind from health programming in Kerala, as well as what was being done to support them and/or address their needs (in general, and in the COVID context).

**Table 1:** Participant characteristics

Category	Designation	Female	Male	Total
Local Self Government Representatives	Panchayat President	3	4	7
	Panchayat Vice-President	0	1	1
	Health Standing Committee Member	3	5	8
	Ward Member	0	1	1
	Community Leader	1	6	7
Health System Actors	Medical Officer	5	3	8
	Health Inspector (HI)	1	5	6
	Public Health Nurse (PHN)	4		4
	Junior Health Inspector (JHI)	0	7	7
	Junior Public Health Nurse (JPHN)	11	0	11
	Nursing Officer	3	0	3
	Palliative Nurse	1	0	1
	Community Health Worker	16	0	16
	**Total Participants**	**48**	**32**	**80**

### Who is left behind?

Participants in all districts would often first identify Scheduled Caste and Scheduled Tribe communities as vulnerable; these are nationally established categories defined as facing vulnerability. Apart from this, we observed geographical variation across districts in who was described as vulnerable population by stakeholders (see Table 2). Migrant labourers were identified as vulnerable in the semi-urban areas, while fisherfolk in the coastal areas (inland and seafaring).

**Table 2:** Vulnerable Population Identified by Participants across Districts

	Thiruvananthapuram	Kollam	Alappuzha	Kasaragod
People from Scheduled Tribe	X		X	X
People from Scheduled Caste	X	X	X	X
Palliative Care patients	X	X		X
Fisherfolk		X (inland)	X (seafaring)	
Farmers			X	X
Migrants			X	X

It was found that most of the places where the vulnerable population were identified, faced challenges related to living and working conditions - social determinants of health like sanitation, nutrition, crowding/housing were raised. According to a Medical Officer,

*…there is the ****SC/ST community****- they have colonies*^1^*here… they have drinking water issues, food issues, improper waste management, and crowded places. It is a dengue hotspot and communicable diseases (hotspot). Also, COVID is a big issue there, because if it affects one person, the spread will be too much…because even the children run around and enter all the houses.*

We also found that climate change (subsequent floods in the state) and COVID-19 pandemic had affected population subgroups and added to their vulnerability. Farm workers were affected by the consequent floods in the state and fisherfolk were affected by the COVID-19 pandemic. One Community Leader noted this:

*…Especially when there were floods*, ***farm workers ****were there…. the one who is mostly engaged with paddy fields. Last financial year was a time when the yield was maximum but there was a technical difficulty in harvesting it. During such a situation, the farmers had to face a lot of trouble.****People turn out to be marginalised when they cannot harvest their crop***. *The situation is similar in the case of ****fisheries*** *as well. Due to COVID, they could not go fishing for several days. Even if they went, there was a situation that people turned COVID positive because there were about 40 people in a fishing boat...*

On the other hand, a few people we spoke to also mentioned that nobody was vulnerable, because the needs of all were catered to, as per need. A Junior Public Health Nurse said: “*I don’t think such a marginalised community exists anymore in this era. We all are equal. I do not think any community is being sidelined nowadays.”*

This view was held by another JPHN as well who took the view that

*There are no marginalised communities in my area. All the people here are from similar backgrounds since it is a coastal area. I do not know if they have any issues. Most of the people over there depend on their daily income and even when they must undergo quarantine, the authorities have delivered them essential commodities and resolved the problems that came up. So, there were no issues, all such troubles were taken care of.*

### Programs to support those left behind

We found that schemes and programmes targeting vulnerable populations were being implemented across the state in most cases. The possible exception we found was the case of fisherfolk and farmers, who were defined as vulnerable, but were not described as being covered by many government health schemes. Recently implemented primary health care reforms had reportedly improved access to healthcare for vulnerable groups in some areas. In many cases this involved interdepartmental coordination. A Panchayat president took the following view:

*Our Family Health Centre works from 7 AM till 8 PM even now. The service of a gynaecology specialist is provided twice a week. Then, we have an eye specialist. We have been getting the services of a physiotherapy specialist. People from the rural areas, including the Adivasi community, were able to benefit from these changes. The Tribal Department has been conducting camps in the places where Adivasis [tribal persons] live*

According to a Health Inspector, there was emphasis placed on going to where communities were to offer them care/support and the role of labour department and private employers in health service delivery:

*We have a lot of migrants around here. The labour office is holding special camps for them. Their employers also sometimes book slots in bulk and get the workers vaccinated. As far as we are concerned, we go to their companies and conduct tests and provide other services there.*

We also found that joint programs implemented by LSGs and the Department of Social Justice, such as the Kudumbasree^2^-self help program for women, as well as programs focussing on the elderly population, migrants, destitute and palliative care patients were intended to increase access to healthcare and to improve quality of life for groups facing these forms of disadvantage. A Health Standing Committee Member added:

*…for palliative patients, we provide support from Panchayat and the FHC. Other than this, we have a scheme called ****Ashraya ****for the destitute. We provide them with kits through Kudumbasree. We have another scheme called ****Santhwanam***. *Under this, through Kudumbasree we conduct an event once a year. Ashraya scheme falls under the ambit of this one. Ashraya is for people with no means of support.*

According to a Community Health worker, the Panchayat placed emphasis on palliation and also on the health and welfare of guest or migrant workers:

*Yes, Panchayat provides it. Even medicines and hospital-related services are arranged by the Panchayat. Similarly, the Panchayat has appointed a nurse for palliative care. We visit their homes along with the palliative nurse and provide all possible services to them. If any ****guest workers ****come here, we treat them like our own people, and both the Panchayat and the FHC provide them with all kinds of assistance.*

This was corroborated by a Panchayat President in another district as well:

*We have proper facilities for ensuring the health of people including migrant labourers. …. Grama Panchayat has facilitated the treatment for numerous cancer patients in the area as well as for those with other related diseases. The area has around 250 palliative patients. We have implemented various programs for helping all such patients.*

There was seen to be, therefore, responsibility taken by local leaders for vulnerable groups and the idea that these were “our own people,” whose needs related to health and beyond, were given due attention.

### COVID Outreach for vulnerable populations

Many study participants felt that during the COVID-19 pandemic and consequent lockdowns, vulnerable populations were prioritised. Various health service design changes were described as being introduced to ensure the delivery of essential health care and related services under the stewardship of LSGs. A Junior Public Health Nurse described them as follows:

*We used to provide food to these side-lined people from the community kitchen, and provide medicines from our Tele-OP [out-patient services], when the first wave of COVID started. When COVID started and there were strict lockdowns, from the side of the health department, every day there was one or two vehicles that were arranged from the side of LSGD and in that vehicle, our staff would take details from each area of the positive cases, and create a calculation on how many of them need medicine, and how many homes we need to put a sticker etc, and both these vehicles would cover two different areas without overlapping and delivered, medicine kit is, NCD medicines and Tele OP medicines everywhere promptly.*

Another Panchayat President noted the greater risks of exposure in certain populations and how they were prioritised commensurably, saying that “*we have distributed kits in every ward. Due to COVID and lockdown, people were not able to go outside so we distributed kits to everyone. We especially distributed masks and sanitisers in the S[cheduled] T[ribe] colonies and other marginalised colonies. Because they were residing in a densely populated area and there is a high chance of spreading, we provided the kits.”*

A Nursing Officer also noted the role played by panchayat leaders in mobilising support during lockdowns, *“when migrants could not go back to their homes, volunteers intervened and helped them. Whatever needed, from* ***food to shelter*** *was provided from the side of the Panchayat.”*

Vulnerable populations were prioritized for receiving COVID-19 vaccinations. There were efforts from the health systems and LSGs to deliver vaccines at the doorsteps of these population. A community health worker described how separate, priority vaccination drives were held for fisherfolk, SC and ST groups. She said simply: “*They were given more preference.”* A Medical Officer noted that in their area, SC, ST, persons living with disabilities and migrants were the first to achieve complete vaccination. This was echoed by a frontline worker in another district who noted that

***Bedridden patients*** *were given vaccination doses at their houses.* ***Palliative patients*** *were given the vaccination at their places. We have also vaccinated people above ****80 years of age*** *after visiting their houses. We visited the houses of ****those who cannot come ****and got them inoculated. We also conduct ****health camps in colonies***. *A class on vaccination programs was also given for them and all these were organised by the PHC.*

## Discussion

Our study sought to identify who was defined as vulnerable by health system and LSG actors in the state of Kerala and what schemes and arrangements were in place to address their health issues. In the current study, we observed that a number of groups identified at the national level as vulnerable were also identified by our study participants, alongside other population groups that were uniquely identified in Kerala. This is consistent with the findings of Kerala State Poverty Eradication Plan presented to NITI Aayog, which reported that SC populations were concentrated in colonies (including in urban areas), ST populations continued to be sequestered in remote and rural locations, consistent with nationally identified groups in need [29]. However, this report also indicated the need to support coastal populations like fisherfolk who for economic reasons were also confined to particular, hard to reach geographies [29]. Decentralized planning in Kerala has helped keep the issue of inclusion and marginalisation on the agenda of decision-makers and implementers, even as newer groups facing vulnerability were being identified, like migrant workers [11]. Migrant workers also faced confinement in their work settings, while palliative care patients were confined due to their health situation. This distance – physical or social – was a defining feature of vulnerability from the perspective of these supply side actors. This kind of a distance based vulnerability has been found in a national studies from Uttar Pradesh, Madhya Pradesh, Bihar Assam and Jharkhand during pre and post COVID-19 periods [30], although the view of health system actors or decision-makers on this was not specifically indicated in the literature. Other studies in LMICs have identified vulnerability on the basis of racial, ethnic and gender minoritization, economically disadvantage, having chronic health issues, as well as those at extremes of age [1, 31, 32]

It was also observed that it was not merely in the context of health, but the larger social determinants that vulnerable populations were “hard to reach.” The residential areas of the marginalized population were underdeveloped: providing quality health service delivery remained challenging without addressing the social determinants of health. This is consistent with the findings of the 6th Kerala Administrative Reforms Commission report (2020) which noted lack of land, improper housing, inadequate infrastructure, poor quality of education, lack of sanitation services and unsafe drinking water among the marginalized population [33]. This report also gave special emphasis on the condition of SC and other “backward” communities who continue to live and work in highly dangerous and pathogenic conditions [33]. It has been deemed vital to address social determinants among the marginalized to improve their health status as they are important factor in management and prevention of communicable and non-communicable diseases alike [34]. Studies conducted in LMICs have reported lower access to safe drinking water, sanitation, and hygiene (WASH), conditions which are fundamental to living and working, are both reflective of vulnerability and are what drive disparities in health burdens, health seeking, and health outcomes [35–37]

We found that natural disasters (floods) and COVID-19 pandemic added to the vulnerabilities faced by farmers and fisherfolk, suggesting that vulnerability is not a static phenomenon. A study conducted by a panel of experts in Kerala immediately after the 2018 floods reported that the vulnerable population who were the victims of floods lagged behind their peer groups in levels of human development, in part because they faced differential and layered exposures and vulnerabilities compared to other groups [38]. Another study by the Palliative Care Consortium on the effect of 2018 floods on elderly living alone found serious after effects of the disaster especially among the elderly women, also the palliative care services and medications were disrupted [39]. COVID-19 lockdowns imposed by the Government during the first wave (2020) affected the coastal community in the state in accessing healthcare and in resourcing the essential commodities. Along with it the declaration of some of the overcrowded coastal regions as containment zones, with restriction of movement leading to reduced working hours and income further increased their vulnerability [40]. A study conducted by Kattungi et al. (2020) assessing the impact of COVID-19 on the livelihood of fishermen in Puducherry found loss of employment among many fishermen which has resulted in increasing inequities and poverty [41]. Aura CM et al. (2020), in their study which assesses the consequences of flooding and COVID-19 Pandemic among inland fisherfolk in Kenya in East Africa, found that natural calamities and pandemic affected the livelihood of fisherfolk, reduced fishing time and trips, decline in consumables such as boat fuel resulting low fish catches etc [42, 43]. COVID- 19 has negatively affected small scale farmers in LMICs which resulted in low production, low income and higher food insecurity which has increased their vulnerability [44, 45]

There has been a fairly high degree of multisectoral action and coordination to reaching the “vulnerable” in Kerala. We found a fascinating convergence in the views of those who identified vulnerable groups and those who did not. Both noted that schemes existed and that vulnerable groups (or everyone!) were taken care of the state through schemes implemented by government departments. This includes multisectoral action led by the State government in prevention and control of Non-communicable Diseases (NCDs) [46, 47], convergence to support awareness of and enrolment in the Department of Labour’s health insurance scheme (supported greatly by LSG leaders and Kudumbasree mission workers under Department of Social Justice), [48]. as well as other schemes introduced by the Kerala Social Security Mission [49–51]

The state’s response in handling the COVID-19 pandemic was another example of multi-sectoral coordination backed by decentralized governance, along with whole of society approaches where community action complemented the work of health system actors [52, 53]. During COVID-19, a community kitchen initiative was introduced through LSGs with the support of Kudumbasree, which provided free meals to labourers, people who were under quarantine, the destitute and other needy marginalized population [54]. Grassroots agencies were also involved with delivering free food kits universally, which required a special focus on vulnerable population typically excluded from social security benefit programmes like transgender persons [53]. In a scoping review by Hasan et al. (2021) about the response of LMICs in management of COVID-19 found that decentralized governance coupled with stewardship and multisectoral collaboration facilitated the delivery of integrated health service delivery[55] ,which was found through our study in Kerala.

Another interesting feature in Kerala was seen during COVID-19 in the context of vaccination. Initially COVID-19 vaccination in Kerala followed global norms by prioritising health workers followed by frontline workers [56], then national norms prioritising citizens above the age of 60 years and citizens aged between 45 and 59 with specified comorbidities [57]. However, by April 2021 Kerala created state specific norms by way of 32 priority categories in the age group of 18–45 which included other frontline workers, seafarers, field staff, teachers, students and more [58]. This demonstrates the possibility of defining and redefining those in need in the context of a crisis. It is less clear, however, if such prioritization of populations in need could be done on an ongoing basis, helping the state to identify those who may face unique disadvantages and may need to be reached by programming beyond the existing ambit. This is a clear area for further research.

Beyond this, there are other areas warranting further research: greater attention to how multi-sectoral policy processes for the “vulnerable” take place, in what contexts, could offer lessons for their replication in other contexts, and also for their enhancement in Kerala. Moreover, it is unclear, at present, how intersections of vulnerability may be addressed in current programming, for e.g. SC or ST populations receiving palliative care, women involved with the fishing industry. Whether or not such programs are catering to these intersectional needs would be a critical area for future policymaking. Finally, there is a very little understanding of those facing vulnerability as being more than “target populations” or “beneficiaries” of services. Other research on UHC has shown that just producing interventions and considering communities passive recipients can easily alienate and exclude them from health reform processes[59]. Further study is needed – across all these and more groups facing vulnerability – on how they perceive themselves, and how they receive, and experience schemes designed for them, and in the absence of such schemes, how they manage their health and related needs. This would have to be given more attention in research and policymaking and is a limitation in the framing of our study as well.

### Limitations

This analysis is based on the perceptions of government health system actors. It therefore does not include the perceptions of the general population as well as those who constitute “those left behind.” Research is currently underway to understand the care seeking experiences of these, “demand side” actors and is a crucial part of our understanding of vulnerability.

## Conclusion

Our analysis sought to understand supply side perspectives in the health sector on who is left behind in the southern Indian state of Kerala. Health system actors and local self-government members were aware of vulnerable population prioritized under various schemes but did not describe vulnerable groups beyond this. Emphasis was placed on the broad range of services available to these “left behind” groups. Further study (currently underway) may offer insights into how these communities – identified as vulnerable – perceive themselves, and how they receive, and experience schemes designed for them. Innovative sampling and recruitment mechanisms need to be devised to identify populations who are currently left behind but may also be invisible to system actors and leaders.

While the Kerala government has shown initiative in carrying out a mapping of poorest households in the state, there are other critical forms of vulnerability that affect residents in the state; continuous monitoring of “who is being left behind,“ in partnership with academic and civil society institutions, could help enhance such initiatives.

## Data Availability

All datasets used for supporting the conclusions of this paper are available from the corresponding author on request.

## Footnotes

1 While system actors often mentioned colonies of SC and ST communities, in subsequent fieldwork, SC communities in particular felt offended by the label of “colony” used to describe their places of residence. This could be seen as being akin to what Wacquant has called “territorial stigma,” which automatically assigns ignominy to a geographic category.(27) Although Wacquant’s theorization referred to the urban context in Chicago and Paris alone, we saw resonance of the concept for urban and rural residents of “colonies.” The concept of the “colony,” of course, has other problematic histories and legacies.

2 Kudumbashree is the poverty eradication and women empowerment programme implemented by the State Poverty Eradication Mission (SPEM) of the Government of Kerala.[28]. More information is available at: https://www.kudumbashree.org.

## List of abbreviations

SDGs: Sustainable Development Goals
LNOB: Leave No One Behind
SC: Schedule Caste
STs: Schedule Tribes
OBCs: Other Backward Castes
EBCs: Economically Backward Castes
SKs: Safai Karmacharis
BPL: Below Poverty Line
AB-PMJAY: Ayushman Bharat Pradhan Mantri Jan Arogya Yojana
MGNREGA: Mahatma Gandhi National Rural Employment Guarantee Act
OOPE: Out-of-Pocket Expenditure
PHCR: Primary Health Care Reform
IDIs: In-depth Interviews
HSAs: Health System Actors
FHC: Family Health Centre
LSG: Local Self-Government
MO: Medical Officer
HI: Health Inspector
JHI: Junior Health Inspector
PHN: Public Health Nurse
JPHN: Junior Public Health Nurse
ASHAs: Accredited Social Health Activists
PIS: Participation Information Sheet
